# Identification of Bottle Gourd (*Lagenaria siceraria*) *OVATE* Family Genes and Functional Characterization of *LsOVATE1*

**DOI:** 10.3390/biom13010085

**Published:** 2022-12-30

**Authors:** Zishan Feng, Xiaohua Wu, Jian Wang, Xinyi Wu, Baogen Wang, Zhongfu Lu, Zihong Ye, Guojing Li, Ying Wang

**Affiliations:** 1 College of Life Sciences, China Jiliang University, Hangzhou 310018, China; 2 Institute of Vegetables, Zhejiang Academy of Agricultural Sciences, Hangzhou 310012, China; 3 State Key Laboratory for Managing Biotic and Chemical Threats to the Quality and Safety of Agro-Products, Zhejiang Academy of Agricultural Sciences, Hangzhou 310012, China

**Keywords:** bottle gourd, *OVATE* gene family, fruit shape, *LsOVATE1* gene, gene function

## Abstract

The *OVATE* gene family is a class of conserved transcription factors that play significant roles in plant growth, development, and abiotic stress, and also affect fruit shape in vegetable crops. Bottle gourd (*Lagenaria siceraria*), commonly known as calabash or gourd, is an annual climber belonging to the Cucurbitaceae family. Studies on bottle gourd *OVATE* genes are limited. In this study, we performed genome-wide identification of the *OVATE* gene family in bottle gourd, and identified a total of 20 *OVATE* family genes. The identified genes were unevenly distributed across 11 bottle gourd chromosomes. We also analyzed the gene homology, amino acid sequence conservation, and three-dimensional protein structure (via prediction) of the 20 *OVATE* family genes. We used RNA-seq data to perform expression analysis, which found 20 *OVATE* family genes to be differentially expressed based on spatial and temporal characteristics, suggesting that they have varying functions in the growth and development of bottle gourd. In situ hybridization and subcellular localization analysis showed that the expression characteristics of the *LsOVATE1* gene, located on chromosome 7 homologous to *OVATE*, is a candidate gene for affecting the fruit shape of bottle gourd. In addition, RT-qPCR data from bottle gourd roots, stems, leaves, and flowers showed different spatial expression of the *LsOVATE1* gene. The ectopic expression of *LsOVATE1* in tomato generated a phenotype with a distinct fruit shape and development. Transgenic-positive plants that overexpressed *LsOVATE1* had cone-shaped fruit, calyx hypertrophy, petal degeneration, and petal retention after flowering. Our results indicate that *LsOVATE1* could serve important roles in bottle gourd development and fruit shape determination, and provide a basis for future research into the function of *LsOVATE1*.

## 1. Introduction

Bottle gourd [*Lagenaria siceraria* (Mol.) Standl.] (2n = 2x = 22), a member of the Cucurbitaceae family, also known as calabash or long melon, is a cultivated vegetable, medicinal plant, and grafting rootstock [1]. It originated in Africa and was independently domesticated in Asia [2]. With a cultivation history of 7000 years in China, it is one of the characteristic summer vegetables in southern China. Bottle gourd exhibits high genetic variability, especially in fruit shape, which can be round, oblate, pyriform, Hulu (double gourd), dipper, slender straight, tubby, etc. [3,4]. The diverse morphology of the bottle gourd fruit makes it a good candidate for studying variations in fruit shape. Fruit shape is a major horticultural trait for many fruit and vegetable crops that can influence their yield and quality from a crop-breeding perspective [5,6]. It is usually evaluated in terms of fruit diameter (FD), fruit length (FL), and fruit shape index (FSI, the ratio of FL to FD) [7,8]. Changes in fruit shape are not only the result of natural selection but also of artificial domestication to adapt to diverse environments or consumer preferences [9].

Fruit shape has historically been a research hotspot [10,11,12], and many fruit shape-determining genes have been identified or cloned in various crops [13,14,15,16,17,18,19,20]. In tomato, *OVATE* is expressed during flower and fruit development. A single mutation in *OVATE* leads to a premature stop codon, causing tomato fruits to transition from round to pear-shaped [14]. *SUN*, one of the major genes causing elongated fruit shapes in tomato, was found to encode a member of the IQ67 domain-containing family [15]. Research shows that extreme fruit sizes are the result of a regulatory change of a transcription factor (*FASCIATED*) that controls carpel number during flower or fruit development [16]. In addition to *FASCIATED*, a single nucleotide mutation of *LC* will also cause changes in the number of locules [17]. Regarding cucurbit crops, studies have generally focused on cucumber. *SF1* induces larger but fewer fruit cells, which leads to a short fruit phenotype. In addition, transcriptomic analysis revealed that *SF1* might control fruit length via fine-tuning of cytokinin and auxin signaling, gibberellin biosynthesis, and signal transduction in cucumber fruits [18]. *CsCLV3* is a homolog of the Arabidopsis *CLAVATA3* gene. *CsCLV3* induces carpel number variations in cucumber [19]. Further research also showed that *CsCLV3* and *CsWUS* function as a negative and positive regulator for carpel number variation, respectively. Biochemical analyses indicated that *CsWUS* directly binds to the promoter of *CsCLV3* to activate its expression [20].

Among the known fruit shape-related tomato genes, *OVATE* is a representative gene that is also known to affect fruit shape in other crops. Eighteen *OVATE* family transcription factors were identified in *Arabidopsis thaliana*. *AtOFP1* overexpression leads to silique shortening. Furthermore, this gene inhibits cell elongation by inhibiting *AtGA20ox1* expression, which encodes gibberellin synthase [21,22]. Rice also contains multiple *OVATE* family transcription factors. The overexpression of *OsOFP1* and *OsOFP2* results in dwarf plants with thicker leaves, anther abortions, and low seed-setting rates [23,24]. In addition, the expression of *OVATE* homologs has been confirmed to be closely related to fruit shape in pepper, peach, and melon [6,25,26,27]. The mechanism by which *OVATE* regulates fruit shape is related to its interaction with *TONNEAU1* Recruiting Motif (*TRM*) family proteins, which are involved in regulating microtubule arrangement in order to control cell division patterns and growth [26,28,29]. In *Arabidopsis thaliana*, TRM proteins (LONGIFOLIA1 (LNG1) and LONGIFOLIA2 (LNG2)) regulate the longitudinal elongation of cells. Overexpression of these two proteins causes the aerial portions of plants, such as leaves, floral organs, and siliques, to become longer, while the cotyledons and pods become shorter if loss-of-function mutations of these proteins occur [30,31]. In tomato, *SlTRM5* knockout in *OVATE*/*SOV1* double mutants induced fruit shapes to change from long pear shapes to round [32].

In previous studies, we performed RAD-Seq genotyping on a natural population of bottle gourd comprising 80 accessions, based on which two sub-gene pools were suggested to be associated with fruit shape. Outlier testing suggested a locus on LG7, which harbors an ortholog of the tomato fruit shape gene *OVATE*, to be a candidate site for selection [4]. In this study, bottle gourd *OVATE* gene family members were first identified at the whole-genome level. A phylogenetic tree was constructed to assess the evolutionary relationships of 20 *OVATE* family genes between bottle gourd and other plants. Then, we analyzed the conserved amino acid sequences and three-dimensional protein structures of 20 *OVATE* family genes. In addition, the expression patterns of 20 *OVATE* family genes in the ovaries were also analyzed across various developmental stages. We cloned the *OVATE* ortholog named *LsOVATE1*. In situ hybridization, subcellular localization, and RT-qPCR were used to analyze the temporal and spatial expression characteristics of *LsOVATE1.* RNA in situ hybridization analysis revealed that *LsOVATE1* transcripts were detectable in young ovaries, which were largely restricted to the placental area. Finally, ectopic expression of *LsOVATE1* in tomato-induced phenotypes which included cone-shaped fruit, calyx hypertrophy, petal degeneration, and petal retention after flowering. Function analyses suggested a potential regulatory mechanism of *LsOVATE1* during bottle gourd development.

## 2. Materials and Methods

### 2.1. Plant Materials

In this study, plant materials included the ‘Hangzhou gourd’ (hereafter ‘HZ’) and YD-4 (Figure S1). HZ is a local cultivar with slender straight fruit, which originated from Southeast China. YD-4 is a landrace with near-round fruit. For *OVATE* gene family member expression profiling, roots, stems, leaves, male flowers, female flowers, immature fruits, and ovaries (8 days before anthesis [DBA], 6 DBA, 4 DBA, and 0 DBA) from HZ and YD-4 were used. Ten individuals of each accession were grown in 30 m rows, spaced 0.5 m apart, at the Haining experimental station (30° N, 120° E).

### 2.2. Identification and Phylogenetic Analysis of the OVATE Gene Family 

For identification of the *OVATE* gene family, the sequences of tomato OVATE proteins were obtained from the Sol Genomics Network (https://www.sgn.cornell.edu/, accessed on 20 March 2020). These sequences were used as queries to search against bottle gourd proteins using the BLASTP program with a threshold e-value of 1 × e^−50^. Data on *OVATE* family genes in bottle gourd were downloaded from the Cucurbit Genomics Database (http://cucurbitgenomics.org/, accessed on 20 March 2020) and Gourdbase (http://www.gourdbase.cn/, accessed on 20 March 2020). The genome data of *Arabidopsis thaliana* were downloaded from TAIR (https://www.arabidopsis.org/, accessed on 20 March 2020). Phylogenetic and molecular evolutionary analyses were conducted using MEGA version 7, based on OVATE protein alignment [33,34]. The phylogenetic tree was constructed in Toolbox for Biologists software (TBtools, Ver. 1.098693) using the maximum likelihood (ML) method with 10,000 bootstrap number.

### 2.3. Sequence Alignment, Chromosomal Location, and Three-Dimensional Protein Structure Prediction 

*OVATE* gene family members were mapped on bottle gourd chromosomes according to positional information from Gourdbase. BioEdit software [35] was used to compare the homologous proteins between bottle gourd and tomatoes (using amino acid sequence information), and data of the conserved domain were analyzed. The online SWISS-MODEL software (https://swissmodel.expasy.org/, accessed on 25 March 2020), which can predict the three-dimensional structure of proteins upon input of the amino acid sequence of related proteins, was used to predict the three-dimensional structures of homologs in bottle gourd. These tools were used to compare the differences in genes in the *OVATE* family.

### 2.4. RNA Extraction and RT-qPCR Analysis

Total RNA was extracted using TRIzol Reagent (Plant RNA Purification Reagent for plant tissue) according to the manufacturer’s instructions (Invitrogen, Carlsbad, CA, USA). Reverse transcription was performed using SynScipt™ III cDNA Synthesis Mix (Tsingke, Beijing, China) with 1 µg total RNA. The cDNA samples were stored at −20 °C before being used. Gene expression was examined via RT-qPCR using the SYBR Green method on a StepOne^TM^ real-time PCR System. PCR amplifications were performed on a StepOne^TM^ real-time thermal cycler in a final volume of 20 µL containing 1.0 µL of cDNA, 0.8 µL of each primer (10 µM), 7.0 µL of sterile water, and 10 µL (2×) of TSINGKE Master qPCR Mix (SYBR Green I) (Tsingke). The amplification conditions were as follows: 1 min of denaturation at 95 °C, followed by 40 cycles at 95 °C for 10 s, 60 °C for 30 s, and 72 °C for 30 s, after which a melt curve was produced at 60 °C. Relative gene expressions were estimated according to the 2^−∆∆Ct^ method [36,37]. The bottle gourd TuB-α (tubulin alpha chain-like) gene (*HG_GLEAN_10019204*) was used as a reference gene. Primer sequences of the reference and target genes are listed in Table S1. Three replicates were performed for each reaction. The relationship between gene expression and phenotype was further obtained by analyzing gene expression using RT-qPCR.

### 2.5. Transcriptional Profiling

To intuitively compare the expression differences of 20 *OVATE* family genes in different fruit types and different periods, an Agilent 2100 Bioanalyzer (Agilent Technologies, Santa Rosa, CA, USA) was used to assess the quality of the total RNAs and RNA samples with RNA integrity numbers > 7 were selected for library preparation. Multiplexed libraries for next-generation sequencing were prepared using the TruSeq RNA Sample Prep Kit v2 (Illumina, San Diego, CA, USA) according to the manufacturer’s instructions. Paired-end sequencing was performed using an Illumina HiSeq 4000 platform. The raw paired-end reads were trimmed and quality controlled using SeqPrep (https://github.com/jstjohn/SeqPrep, accessed on 1 July 2020) and Sickle (https://github.com/najoshi/sickle, accessed on 1 July 2020) with the default parameters. TopHat (v2.1.0) was used to align the high-quality reads to the reference genome. The count of the mapped reads from each sample was derived and normalized to reads per kilobase of exon per million mapped reads (RPKM) for each predicted transcript using Cufflinks (v2.2.1). [38,39].

### 2.6. In Situ Hybridization

Young ovaries at 8, 4, and 0 DBA from HZ and YD-4 were fixed in 3.7% formol-acetic-alcohol and stored at 4 °C until use. In situ hybridization was performed as previously described [40]. The *LsOVATE1* probe was designed according to the specific gene fragments. Sense and antisense probes were generated via PCR amplification with specific primers using SP6 and T7 polymerase, respectively. The primers for probe synthesis are listed in Table S1. 

### 2.7. Subcellular Localization

The *LsOVATE1* probe was inserted into the N-terminus of green fluorescent protein (GFP) in a pCAMBIA1305-GFP vector, generating the fusion construct *p35S:LsOVATE1-GFP*. The fusion expression and empty vectors were transformed into DH5α (Tsingke) competent cells, and after PCR amplification and verification by sequencing, these plasmids were transformed into competent *Agrobacterium tumefaciens* GV3101 (Tsingke) cells using the freezing method. The *A. tumefaciens* cells containing the recombinant plasmid and blank control were injected into the abaxial side of tobacco leaf explants using the vacuum infiltration method. The fusion expression vector was transiently expressed in *N. benthamiana* leaves. Fluorescent signals were detected using a laser confocal scanning microscope (ZEISS Microsystems LSM 700) [41].

### 2.8. Construction of Overexpression Plasmid and Phenotypic Evaluation of Transgenic Plants

After the study of gene expression characteristics, the *LsOVATE1* function was further verified through transgenic analysis. The full-length CDS of *LsOVATE1* was PCR-amplified using a cDNA sample prepared from an ‘Hangzhou gourd’ ovary with gene-specific primers (Table S1). The PCR reaction was carried out using kodakarensis (KOD) and DNA polymerase (ToYoBo, Shanghai, China) in a total volume of 50 µL. The procedure was as follows: an initial denaturing step at 95 °C for 5 min, 34 cycles of 95 °C for 30 s, 58 °C for 30 s, and 68 °C for 90 s, and a final extension step at 68 °C for 7 min. The *LsOVATE1* CDS was inserted into the BamHI and SpeI sites of the pCAMBIA1305-GFP vector. The primer sequences used to obtain the target gene and overexpression vector are listed in Table S1. The constructs were then transformed into tomato using *A. tumefaciens* GV3101. Tomato transformation was performed as previously described [42]. Transgenic tomatoes were verified with PCR using the vector- and gene-specific primers listed in Table S1. Wild tomatoes were used as control plants.

## 3. Results

### 3.1. Identification of OVATE Family Genes in Bottle Gourd

To identify *OVATE* family genes in the bottle gourd genome, BLAST searches were performed using tomato OVATE proteins as query sequences. The sequences of candidate proteins identified from the BLAST searches were compared. Sequences encoding very short polypeptides or those that did not contain the conserved motifs (characteristic of *OVATE* family proteins) were excluded after phylogenetic and conserved domain analysis. After multiple screening and validation steps of the conserved domains, we finally identified 20 putative *OVATE* genes (Table 1). To determine the chromosomal distribution of the identified bottle gourd genes, their physical locations on the bottle gourd chromosomes were investigated. As result, the 20 genes were mapped on 11 chromosomes. Chromosomes 1, 2, 6, 7, 9, 10, and 11 contained one gene each; chromosomes 8 contained two genes each; and chromosome 4 and 5 contained three genes. Five *OVATE* homologs were located on chromosome 3, which is the chromosome with the maximum number of homologous genes.

### 3.2. Phylogenetic Relationship of the OVATE Family Genes in Bottle Gourd

**Figure 1:**
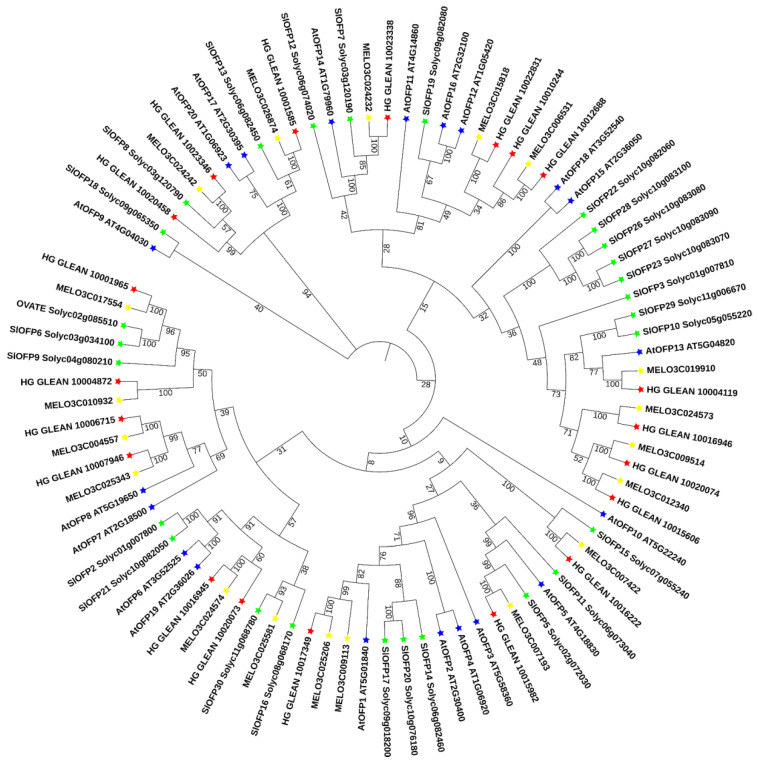
Phylogenetic relationships of the *OVATE* family in plants. The phylogenetic tree was constructed using full-length amino acid sequences of the 86 *OVATE* gene families and MEGA 7.0. Green, yellow, red, and blue stars represent tomato, melon, bottle gourd and Arabidopsis genes, respectively.

To evaluate the evolutionary relationships among OVATE proteins, a phylogenetic tree was constructed using the protein sequences from the 20 putative bottle gourd *OVATE* gene families, 19 melon *OVATE* gene families, 20 Arabidopsis *OVATE* gene families, and 27 tomato *OVATE* gene families, including *OVATE* (Figure 1). Specific information on the other *OVATE* gene families is provided in Table S2. The evolutionary tree classified the *OVATE* genes into four clades with well-supported bootstrap values. The results show that bottle gourd *OVATE* genes were unevenly distributed in three clades, with the gene related to bottle gourd shape (chromosome 7) having high homology with the tomato *OVATE* gene. The corresponding GO analysis is shown in Table S3.

### 3.3. Structural Characterization of Bottle Gourd OVATE Family Genes

**Figure 2:**
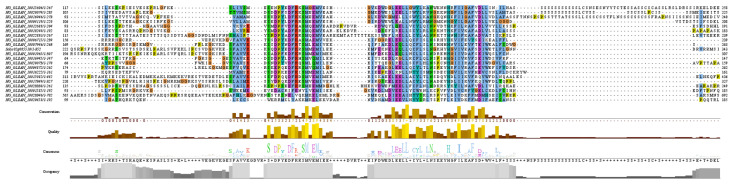
Sequence alignment of OVATE homologous proteins between bottle gourd and tomato.

**Figure 3:**
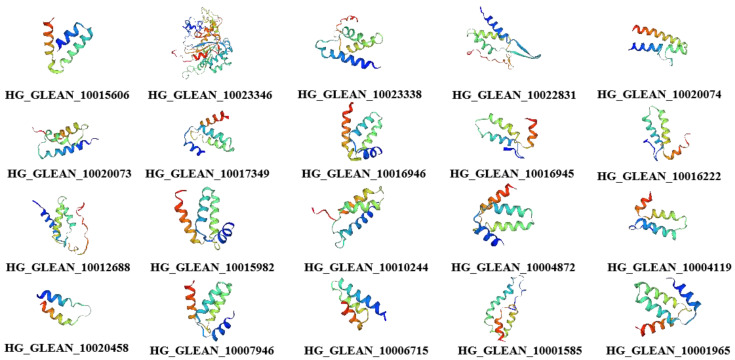
Predicted three-dimensional structures of bottle gourd OVATE family proteins.

The structures of the proteins encoded by the 20 *OVATE* family genes in bottle gourd were predicted, and the amino acid sequences of the OVATE proteins were aligned with the tomato OVATE protein (Figure 2). An analysis of the conserved domains of the bottle gourd *OVATE* family genes revealed that corresponding identical amino acids were found at multiple sites. This suggests that the 20 identified genes have similar structures and functions, and are also similar to the tomato *OVATE* gene. This result indicates that the *OVATE* gene family is highly conserved among these species. The three-dimensional protein structure predictions showed that bottle gourd OVATE family proteins were mainly composed of α-helices and random coils (Figure 3). In terms of spatial configuration, *HG_GLEAN_10017349*, *HG_GLEAN_10016946*, *HG_GLEAN_10015982*, *HG_GLEAN_10004872*, *HG_GLEAN_10007946,* and *HG_GLEAN_10001965* showed high similarity. Among the 20 genes, only *HG_GLEAN_10023346* contains introns, likely because the three-dimensional structure of its encoded protein has the highest complexity.

### 3.4. Expression Profiles of the OVATE Gene Family in the Bottle Gourd

**Figure 4:**
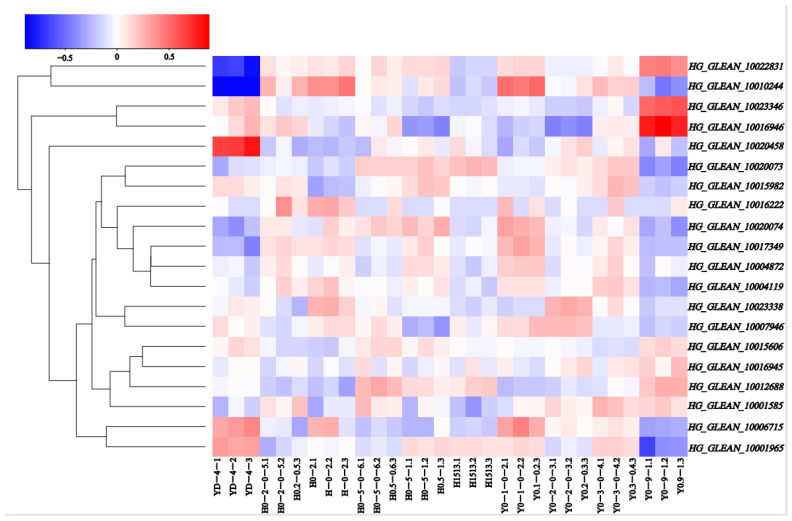
*OVATE* gene family members expression profiles in the ovaries of bottle gourd. Blue or red indicates lower or higher levels of expression, respectively, of each transcript in each sample.

RNA-seq data were used to observe the expression patterns at five stages of ovarian development in both HZ and YD-4 bottle gourd (8, 6, 4, 2, and 0 DBA), in order to assess the possible roles of the 20 *OVATE* family genes in the growth and development of bottle gourd (Table S4). A heat map was constructed according to the Log10 values (tpm + 0.0001) of the genes (Figure 4). The levels of expression varied widely between different bottle gourd *OVATE* family genes, and between different stages in individual bottle gourd. Some *OVATE* family genes in bottle gourd demonstrated opposing expression patterns; for example, *HG_GLEAN_10020458* is highly expressed in YD-4 at 0 DBA, but *HG_GLEAN_10022831* and *HG_GLEAN_10010244* are the opposite. *HG_GLEAN_10016946* is highly expressed in YD-4 at 2 DBA, but *HG_GLEAN_10001965* shows the opposite expression trend. These varying expression levels suggest that these genes may play different significant roles during different developmental stages.

### 3.5. Expression Characteristics of LsOVATE1 Gene 

**Figure 5:**
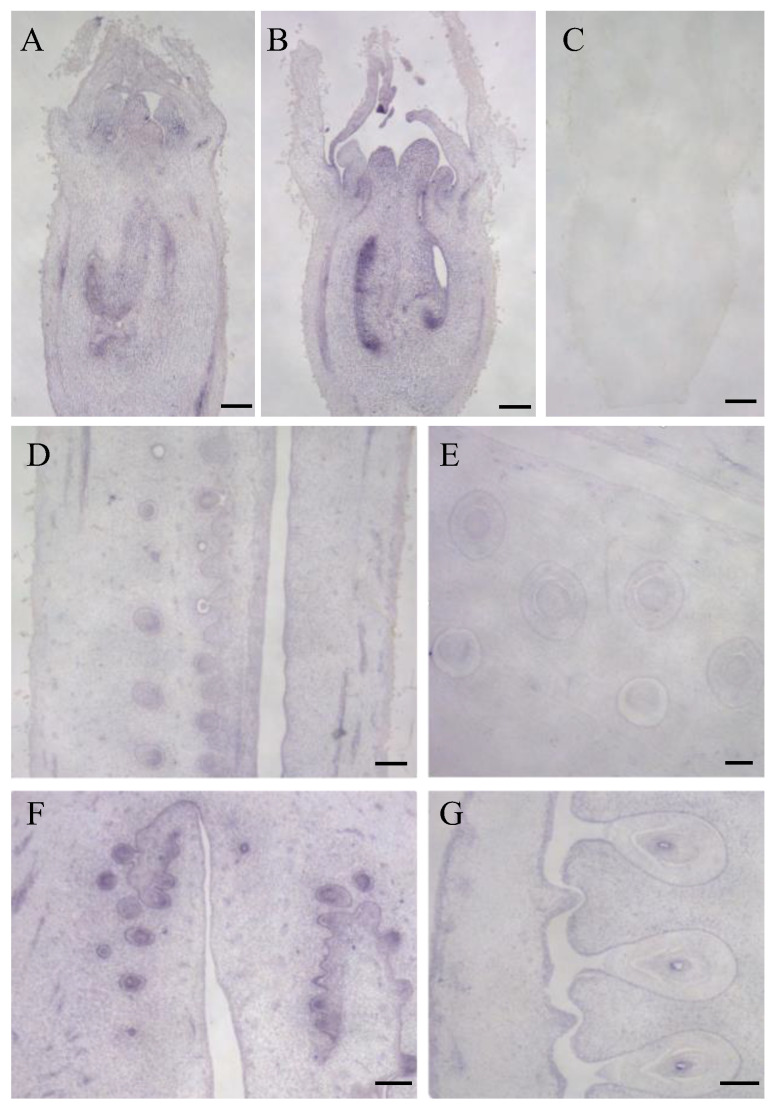
In situ hybridization of *LsOVATE1*. (**A**,**B**) show longitudinal sections of young ovaries at 8 days before anthesis in HZ and YD-4, respectively; (**D**,**E**) show longitudinal sections of young ovaries at 4 days before anthesis in HZ and YD-4, respectively. (**F**,**G**) show longitudinal sections of ovaries on the day of anthesis in HZ and YD-4, respectively. (**C**) indicates that negative controls successfully hybridized with the sense probe. Scale bar = 200 µM.

**Figure 6:**
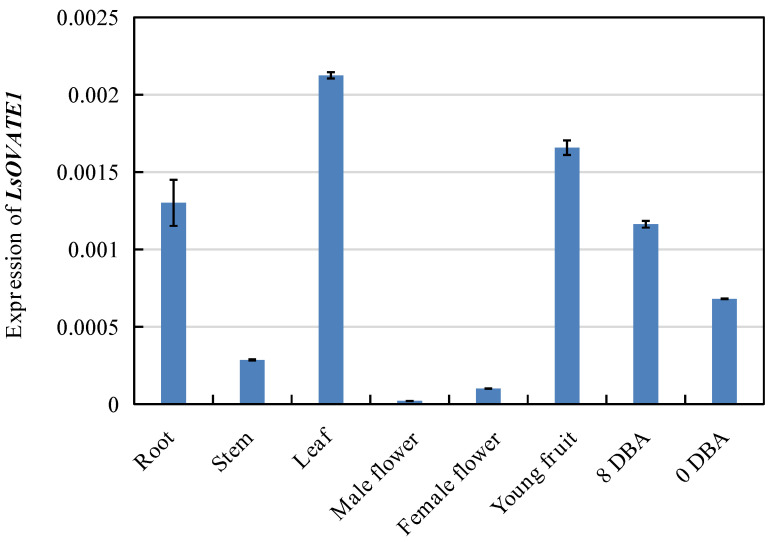
*LsOVATE1* expression levels in different organs and growth stages, including expression of *LsOVATE1* in roots, stems, leaves, male flowers, female flowers, young fruits, and ovaries at 8 days before anthesis (8 DBA) and 0 days before anthesis (0 DBA) from HZ plants. Values are means ± SD of three replicates.

**Figure 7:**
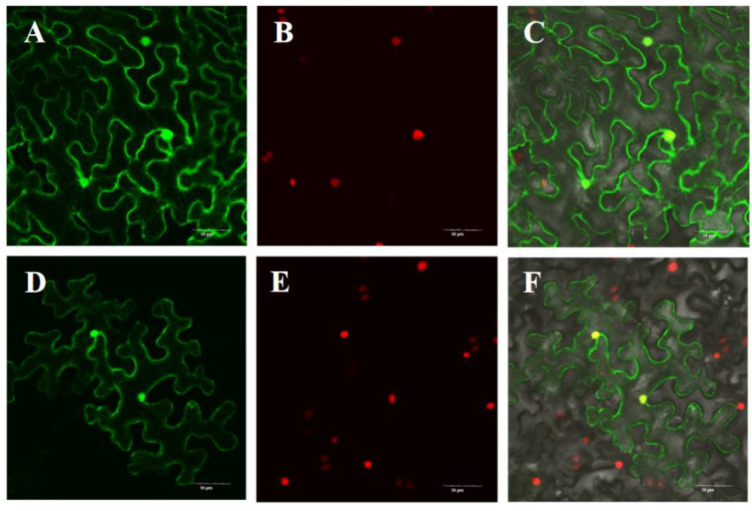
Subcellular localization of *LsOVATE1* in *N. benthamiana* epidermal cells. Empty GFP vectors were used as controls, and showed green fluorescence. The tobacco nuclei showed red fluorescence. C and F are digitally merged images of both bright-field and fluorescent signals. (**A**–**C**) show the localization of empty GFP control vector; (**D**–**F**) show the localization of *LsOVATE1*. Scale bar = 50 µM.

To provide new insights into the expression characteristics of the *LsOVATE1* gene, we first cloned *LsOVATE1* (720 bp) (Figure S2). Next, we performed RT-qPCR and in situ hybridization to analyze *LsOVATE1* expression. Specific RNA probes were used to perform in situ hybridization on HZ and YD-4 samples at 8, 4, and 0 DBA (Figure 5). The results showed that *LsOVATE1* is expressed in tissues around the embryonic seat of young ovaries at the 8, 4, and 0 DBA growth stages. *LsOVATE1* expression at different stages of growth was consistent between HZ and YD-4 specimens. When hybridization was performed with the sense probe (Figure 5C), no signal was detected. In addition, RT-qPCR analysis showed that *LsOVATE1* was most highly expressed in leaves, followed by roots and young fruits, while male flowers had the lowest expression levels (Figure 6). The gene was expressed differently at different growth stages, but no significant expression trend was evident. To analyze the subcellular localization of *LsOVATE1*, *p35S:CmOFP13-GFP* constructs were transfected into *N. benthamiana* epidermal cells for transient expression. We found that the LsOVATE1-GFP fusion protein was localized both in the cytoplasm and nucleus (Figure 7).

### 3.6. Functional Validation of LsOVATE1 

**Figure 8:**
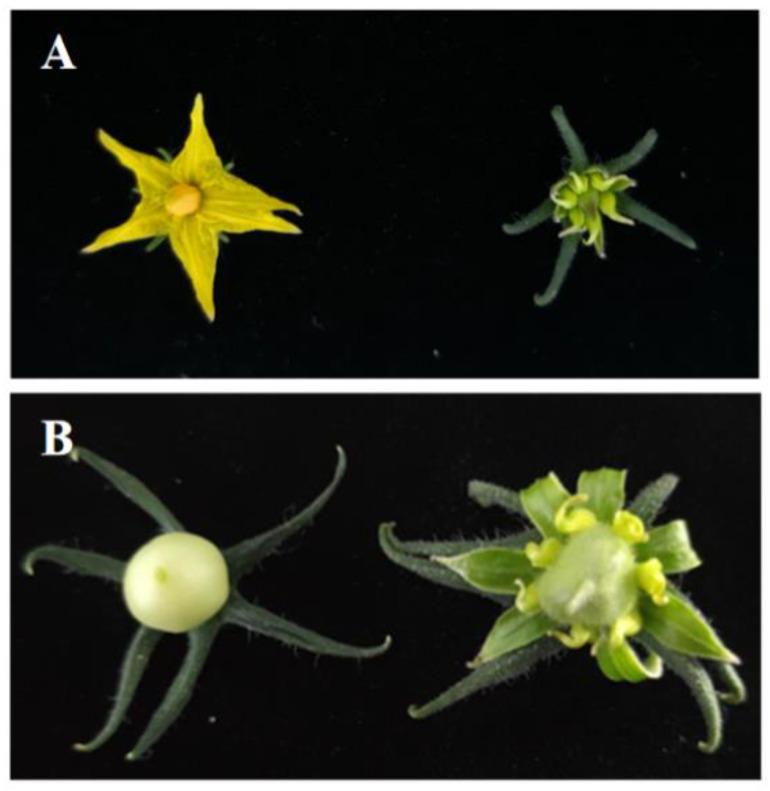
Phenotypes of transgenic plants overexpressing the *LsOVATE1* gene compared to wild-type (WT) tomato. (**A**) shows the flowers of the representative transgenic line (right) and that of the WT (left); (**B**) shows the young fruits of the representative transgenic line (right) and that of the WT (left).

**Figure 9:**
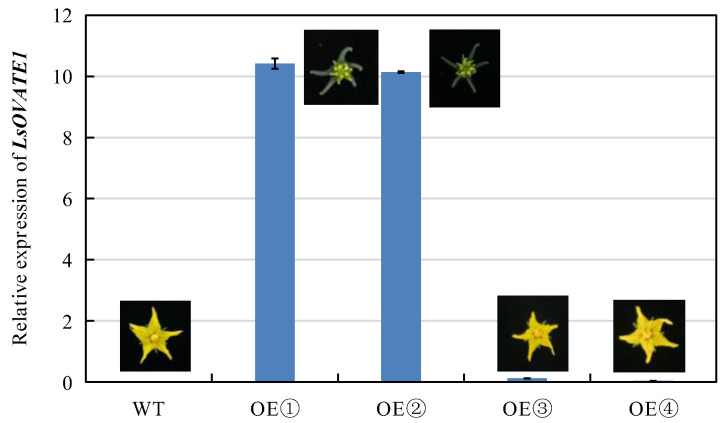
*LsOVATE1* expression levels in overexpressing and wild-type plants. Relative expression of *LsOVATE1* in wild-type (WT) tomato and four overexpressing tomatoes (OE➀, OE➁, OE➂, OE➃). Values are means ± SD of three replicates.

To further validate the function of *LsOVATE1*, we used the CaMV35S promoter to overexpress the coding sequence. Since transformation technology is commonly difficult to use in bottle gourd, we could not confirm the function of *LsOVATE1* by generating transgenic bottle gourd lines. Instead, we transformed the *LsOVATE1* overexpression vector (*p35S:LsOVATE1*) into wild-type tomato. The transgenic tomato plants were successfully detected using PCR, and the transcript levels of representative transgenic lines were confirmed with RT-qPCR. Compared with wild-type tomato, the fruit of the transgenic plants showed significant changes and a cone-shaped morphology. In addition to the fruit phenotype, phenotypic changes including calyx hypertrophy, petal degeneration, and petal retention after anthesis were observed (Figure 8). Our results indicate that *LsOVATE1* not only affected the fruit shape, but also played a role in petal development. The relative expression levels of *LsOVATE1* in the four transgenic plants with respect to the wild-type control were determined. It was found that *LsOVATE1* expression in transgenic plants correlated positively with the phenotype (Figure 9).

## 4. Discussion

The OVATE protein family is unique to plants and includes specific transcription factors that regulate plant growth and development [43]. *OVATE* was first found to be a major QTL in controlling the development of pear-shaped tomato fruit [44]. *OVATE* was first cloned in tomato, and the *OVATE* domain was found to be conserved across tomato, Arabidopsis, and rice [14,22]. It was further confirmed that *OVATE* overexpression can induce the transition from pear-to round-shaped tomato [45]. 

To date, many *OVATE* family genes, in addition to *OVATE*, have been described. Their functions are related to the regulation of fruit shape; however, different phenotypes arise based on which genes are active. When studying the expression characteristics of tomato *OVATE*, subcellular localization revealed that the gene was localized in the nucleus [46]. In addition, *SlOFP20* was shown to be localized in the nucleus and cytoplasm. Phenotypic observation of *SlOFP20*-overexpressing transgenic tomato plants showed that the tomato fruit shape reverted from pear- to round-shaped [26]. Many *OVATE* family genes have also been found in the model crop Arabidopsis. Nine *AtOFPs* have been found to regulate the development of Arabidopsis meristems and leaves. *AtOFP1* transgenic Arabidopsis plants were found to develop slowly, with shorter leaves and anthers, thicker filaments, and fewer seeds. Subcellular localization analysis showed that *AtOFP1* was localized in the nucleus [21]. A total of 31 transcription factors of the *OVATE* family have been detected in rice [24]. The internodes of rice stems are shortened and the leaves become shorter and wider when *OsOFP22* is overexpressed. *OsOFP1* and *OsOFP2* overexpressed transgenic lines were dwarf in size, with thicker leaves and a lower seed setting rate [23]. In melon crops, *CmFSI8/CmOFP13* (a fruit shape-related gene), has been characterized as a homolog to *SlOFP20* and *AtOFP1* [6]. The overexpression of this gene in Arabidopsis led to significantly smaller and curly kidney-shaped leaves, as well as significantly shorter plants. Similar to *SlOFP20*, *CmFSI8/CmOFP13* is localized both in the cytoplasm and nucleus, but its expression is relatively weaker. The protein encoded by *OVATE* in grapes is also localized in the nucleus. The differential expression of *VvOVATE* in different varieties may be an important factor in fruit shape [47]. In round fruit and long fruit pepper, *CaOVATE* was found to negatively regulate the expression of *CaGA20ox1*, resulting in alterations in the gibberellin synthesis pathway, which led to changes in fruit shape from round to oval [25]. 

In this study, 20 *OVATE* homologs were successfully identified in bottle gourd. These *OVATE* homologs were highly conserved with tomato *OVATE*, which is consistent with previous findings regarding *OVATE* family genes in tomato, rice, and other crops. *LsOVATE1*, a homolog with the highest homology to tomato *OVATE*, was successfully cloned. Subcellular localization and in situ hybridization studies indicated that *LsOVATE1* might be involved in the regulation of nuclear gene transcription. In addition, subcellular localization results were also consistent with those of *OVATE* family genes in other crops [48], and the expressed *LsOVATE1* was localized in the cytoplasm and nucleus. The function of *LsOVATE1* was verified using a transgenic approach, which also proved that *LsOVATE1* could affect fruit shape. *LsOVATE1*-overexpressing transgenic seedlings showed obvious changes in fruit shape and flowering period. The transgenic plants exhibited phenotypic characteristics such as calyx hypertrophy, petal degeneration, and petal retainment after anthesis. During further ripening of young fruits, the transgenic fruit dehisced in the pericarp and flowered further at dehiscence. No mature seeds were found in ripe fruits upon opening. This transgenic phenotype is similar to that of *Arabidopsis thaliana* overexpressing *AtOFP1* [43], which affects petals and pollen, as well as the seed-setting rate of mature seeds. Therefore, it is speculated that this gene affects the pollen activity and reproductive mode of plants. Recently, *VvMADS39* was discovered in grapes [49], and is homologous to the *SEP2* gene of Arabidopsis. The heterologous overexpression of *VvMADS39* reduced the fruit and seed size, as well as the seed number in tomato. Research on *VvMADS39* also found that *MADS39* is required for the normal development of the inner three whorls of floral organs as well as the maintenance of floral meristem identity. Further research found that *SlMADS39*, which is homologous to *VvMADS39*, is expressed in tomatoes, and used CRISPER technology to knock out *SlMADS39*. It was observed that the *SlMADS39-*knockout phenotype was similar to that of *LsOVATE1* overexpression, which was characterized in this study. Whether there is an interaction between the LsOVATE1 protein and MAD proteins needs further verification.

Homologs of the tomato *OVATE*, *SUN*, *FASCIATED,* and *LC* family genes have been suggested as candidate genes regulating fruit shape [9,50,51]. Apart from the above-mentioned “classical” genes, recently an ethylene biosynthesis gene (*ACS2*) [52] and FRUITFULL-like MADS-box gene (*FUL1*) [53] in cucumber, an *AP2/ERF* transcription factor gene (*AP2a*) gene and TONNEAU1 Recruiting Motif protein (*TRM5*) in tomato [26,54], as well as an LRR-RLK family gene (*CAD1)* in peach [55], have also been associated with fruit shape. The *CsFUL1* gene directly depresses the expression of the auxin transporters *CsPIN1* and *CsPIN7*, resulting in decreases in auxin accumulation in fruits [53]. In the bottle gourd, despite the number of fsQTLs mapped [56], no fruit shape gene has been identified thus far. Our results also suggest certain *OVATE* family members as candidate fruit shape genes in the bottle gourd.

## 5. Conclusions

We identified a total of 20 *OVATE* family genes in bottle gourd and divided them into four clades to help understand the evolutionary relationships between these genes, which all had at least one conserved domain. Our chromosomal distribution, sequence alignment, three-dimensional protein structure prediction, and exon/intron analyses of the *OVATE* gene family provide a useful basis for understanding the function of the *OVATE* gene family. The expression pattern analysis demonstrated that *OVATE* family genes in bottle gourd were differentially expressed, indicating that they played different roles in the growth and development of bottle gourd. We studied the candidate gene *LsOVATE1*, and found that it is homologous to *OVATE* and affects fruit shape. We also analyzed the expression characteristics of *LsOVATE1* based on in situ hybridization, expression level, and subcellular localization analyses. In situ hybridization further revealed that *LsOVATE1* transcripts were detectable in young ovaries, which were largely restricted to the placental area. Additionally, *LsOVATE1* overexpression in tomatoes caused fruit shape changes, as well as secondary flower and non-seed phenotypes, highlighting *LsOVATE1’s* role in the regulation of plant development (Figure S3). Further studies on fruit shape-regulating genes should provide new gene information that would allow the generation of new bottle gourd varieties with desirable phenotypes. By comparing with other genes that have similar effects on fruit shape, it is speculated that there may be some regulation mechanism, which ultimately led to the effect of *LsOVATE1* on the fruit shape of bottle gourd. This will guide further study on the regulation mechanism of fruit shape in bottle gourd.

## Figures and Tables

**Table 1:** Twenty *OVATE* family genes in bottle gourd.

Gene ID	Chromosome	Start	End	pI	Molecular Weight (kDa)
*HG_GLEAN_10012688*	1	23,417,053	23,417,667	6.34	23.1
*HG_GLEAN_10015606*	2	28,062,254	28,063,057	4.88	30.0
*HG_GLEAN_10015982*	3	1,914,641	1,924,905	9.51	51.7
*HG_GLEAN_10016222*	3	3,622,948	3,623,433	6.82	18.9
*HG_GLEAN_10016945*	3	9,539,752	9,540,195	7.90	16.8
*HG_GLEAN_10016946*	3	9,555,872	9,556,693	4.55	30.6
*HG_GLEAN_10017349*	3	13,489,371	13,490,354	9.80	36.7
*HG_GLEAN_10020458*	4	32,000,706	32,001,644	9.33	22.4
*HG_GLEAN_10020073*	4	28,549,054	28,549,566	9.67	18.9
*HG_GLEAN_10020074*	4	28,561,476	28,562,183	5.15	25.6
*HG_GLEAN_10022831*	5	28,760,144	28,760,947	5.48	29.2
*HG_GLEAN_10023338*	5	33,196,658	33,197,443	8.20	29.6
*HG_GLEAN_10023346*	5	33,258,189	33,258,818	8.70	79.0
*HG_GLEAN_10010244*	6	20,205,559	20,206,137	8.88	21.6
*HG_GLEAN_10006715*	7	21,324,837	21,325,556	9.96	27.3
*HG_GLEAN_10004119*	8	13,885,568	13,886,230	4.66	25.4
*HG_GLEAN_10004872*	8	21,090,898	21,091,833	9.72	30.1
*HG_GLEAN_10001585*	9	18,363,582	18,364,110	9.45	22.9
*HG_GLEAN_10007946*	10	17,697,582	17,698,373	9.78	30.0
*HG_GLEAN_10001965*	11	2,189,256	2,190,179	8.11	35.3

## Data Availability

Not applicable.

## Supplementary Materials

The following supporting information can be downloaded at: https://www.mdpi.com/article/10.3390/biom13010085/s1, Figure S1: Fruit shape pictures of bottle gourd HZ and YD-4; Figure S2: Cloning of *LsOVATE1* and the pCAMBIA1305.1-GFP vector; Figure S3: Framework figure; Table S1: Primer list summary; Table S2: Composition and chromosomal location of the plants; Table S3: GO analysis of OVATE proteins; Table S4: List of RNA-Seq data.
