# Supplementary material for: Identification of Bottle Gourd (Lagenaria siceraria) OVATE Family Genes and Functional Characterization of LsOVATE1
Source: Biomolecules. 2022 Dec 30;13(1):85. doi: 10.3390/biom13010085 (PMC9855390; doi:10.3390/biom13010085)
Supplement: Supplementary file 1 [file biomolecules-13-00085-s001.zip › Figure S1.pdf]

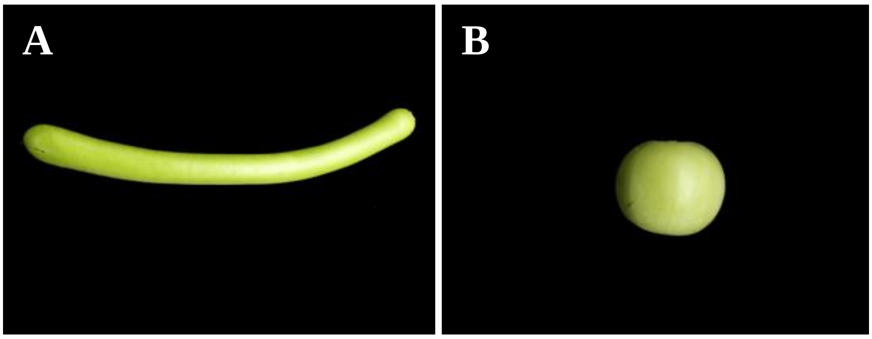

Figure S1. Fruit shape pictures of bottle gourd HZ and YD-4. A is HZ, a local cultivar with slender straight fruit; B is YD-4, a landrace with near-round fruit.
