# Supplementary material for: Identification of Bottle Gourd (Lagenaria siceraria) OVATE Family Genes and Functional Characterization of LsOVATE1
Source: Biomolecules. 2022 Dec 30;13(1):85. doi: 10.3390/biom13010085 (PMC9855390; doi:10.3390/biom13010085)
Supplement: Supplementary file 1 [file biomolecules-13-00085-s001.zip › Figure S2.pdf]

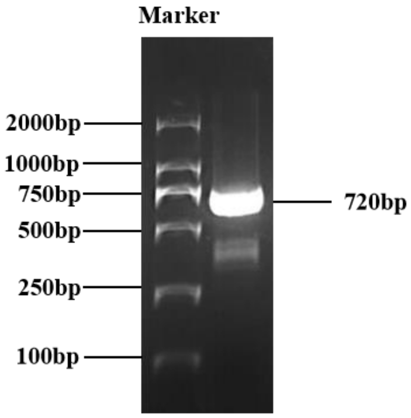

Figure S2. (a) Cloning of *LsOVATE1*. The total length of *LsOVATE1* is 720bp, so the segment between 500-750bp is *LsOVATE1*. Marker: DL2000 (Tsingke, Beijing, China)

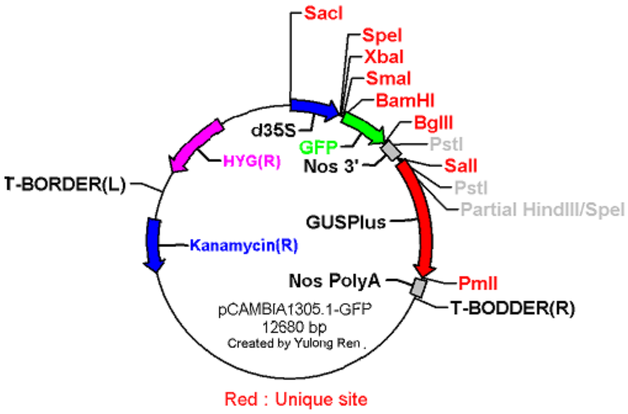

Figure S2. (b) The pCAMBIA1305.1-GFP（Ren et al. 2014）contains a 35S promoter to overexpress the target gene, which is used to observe the phenotype in transgenic plants. The SpeI and BamHI sites on the vector were selected for homologous recombination to obtain the *LsOVATE1* overexpression vector (*p35S:LsOVATE1*).
