# Supplementary material for: Identification of Bottle Gourd (Lagenaria siceraria) OVATE Family Genes and Functional Characterization of LsOVATE1
Source: Biomolecules. 2022 Dec 30;13(1):85. doi: 10.3390/biom13010085 (PMC9855390; doi:10.3390/biom13010085)
Supplement: Supplementary file 1 [file biomolecules-13-00085-s001.zip › Figure S3.pdf]

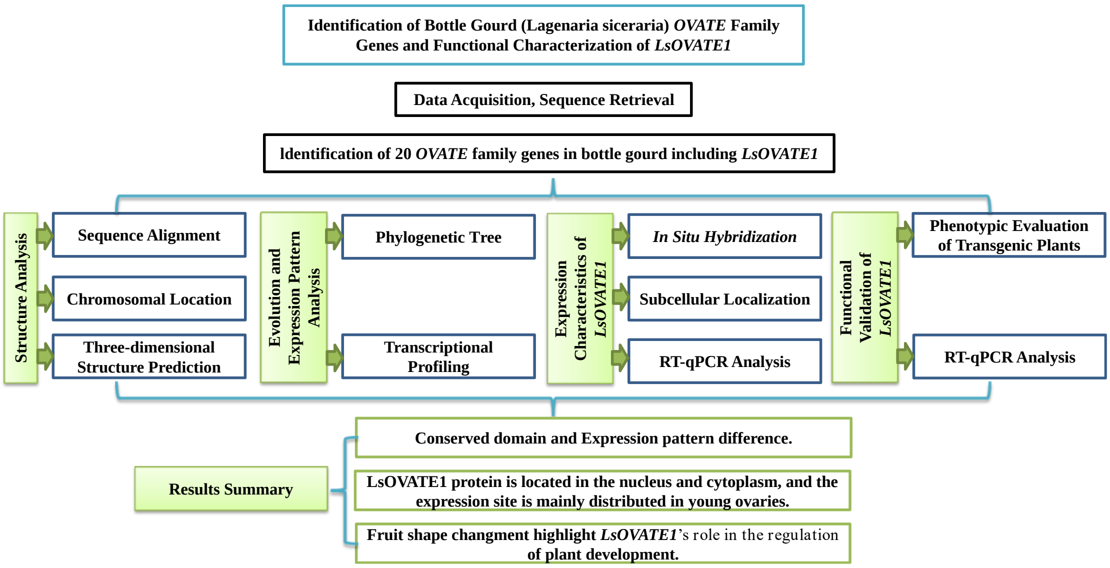

Figure S3. Framework figure. Systematic analysis diagram of *OVATE* gene family in bottle gourd including *LsOVATE1*'s structure, evolution and function.
